# Antibacterial and antibiofilm activity of *Abroma augusta* stabilized silver (Ag) nanoparticles against drug-resistant clinical pathogens

**DOI:** 10.3389/fmolb.2023.1292509

**Published:** 2023-10-30

**Authors:** Sachin Kumar, Haris M. Khan, Fohad Mabood Husain, Rafiq Ahmad, Faizan Abul Qais, Mo Ahamad Khan, Mohammad Jalal, Uzma Tayyaba, Syed Ghazanfar Ali, Amardeep Singh, Mohammad Shahid, Byeong-Il Lee

**Affiliations:** ^1^ Department of Microbiology, J. N. Medical College and Hospital, Aligarh Muslim University, Aligarh, Uttar Pradesh, India; ^2^ Department of Food Science and Nutrition, College of Food and Agriculture Sciences, King Saud University, Riyadh, Saudi Arabia; ^3^ ‘New-senior’ Oriented Smart Health Care Education Center, Pukyong National University, Busan, Republic of Korea; ^4^ Department of Ag. Microbiology, Faculty of Agriculture Sciences, Aligarh Muslim University, Aligarh, India; ^5^ Department of Genetics and Plant Breeding, Chaudhary Charan Singh University, Meerut, Uttar Pradesh, India; ^6^ Department of Microbiology, Immunology and Infectious Diseases, College of Medicine and Medical Sciences, Arabian Gulf University, Manama, Bahrain; ^7^ Industry 4.0 Convergence Bionics Engineering, Pukyong National University, Busan, Republic of Korea; ^8^ Digital Healthcare Research Center, Institute of Information Technology and Convergence, Pukyong National University, Busan, Republic of Korea; ^9^ Division of Smart Healthcare, College of Information Technology and Convergence, Pukyong National University, Busan, Republic of Korea

**Keywords:** silver nanoparticles, green synthesis, antibacterial, *Abroma augusta*, MRSA, VRE, biofilm

## Abstract

Infectious diseases remain among the most pressing concerns for human health. This issue has grown even more complex with the emergence of multidrug-resistant (MDR) bacteria. To address bacterial infections, nanoparticles have emerged as a promising avenue, offering the potential to target bacteria at multiple levels and effectively eliminate them. In this study, silver nanoparticles (AA-AgNPs) were synthesized using the leaf extract of a medicinal plant, *Abroma augusta*. The synthesis method is straightforward, safe, cost-effective, and environment friendly, utilizing the leaf extract of this Ayurvedic herb. The UV-vis absorbance peak at 424 nm indicated the formation of AA-AgNPs, with the involvement of numerous functional groups in the synthesis and stabilization of the particles. AA-AgNPs exhibited robust antibacterial and antibiofilm activities against methicillin-resistant *Staphylococcus aureus* (MRSA) and vancomycin-resistant *Enterococci* (VRE). The MIC values of AA-AgNPs ranged from 8 to 32 μg/mL. Electron microscopic examination of the interaction of AA-AgNPs with the test bacterial pathogens showed a deleterious impact on bacterial morphology, resulting from membrane rupture and leakage of intracellular components. AA-AgNPs also demonstrated a dose-dependent effect in curtailing biofilm formation below inhibitory doses. Overall, this study highlights the potential of AA-AgNPs in the successful inhibition of both the growth and biofilms of MRSA and VRE bacteria. Following studies on toxicity and dose optimization, such AgNPs could be developed into effective medical remedies against infections.

## 1 Introduction

Several pathogenic bacteria have evolved to become the primary culprits behind severe infectious diseases. The issue has grown more complex with the emergence of multidrug-resistant (MDR) bacteria (Hamida et al., 2020). Excluding extensively drug-resistant tuberculosis, the term “multidrug-resistant” pertains to resistance against multiple drugs (Murray et al., 2022). The significant mortality rate observed over the past two decades due to vancomycin-resistant enterococci is worrisome. These resistant bacteria pose a notable threat to current medical procedures as they are resistant to the drugs and are nosocomial infectious agents (Raza et al., n.d.). The six leading pathogens causing deaths related to resistance are *Escherichia coli*, followed by *Staphylococcus aureus*, *Klebsiella pneumoniae*, *Streptococcus pneumoniae*, *Acinetobacter baumannii*, and *Pseudomonas aeruginosa*. In 2019, there were 929,000 (ranging from 660,000 to 1,270,000) deaths attributed to antimicrobial resistance (AMR), with 357 million (ranging from 262 to 478 million) cases associated with AMR-related issues. Among these, methicillin-resistant *S. aureus* (MRSA) was responsible for over 100,000 deaths in 2019 attributed to AMR. Six other variations each resulted in 50,000 to 100,000 deaths (Oves et al., 2023).

The challenge of MDR bacteria is rapidly spreading due to the uncontrolled and indiscriminate use of antibiotics for bacterial infections (Yah and Simate, 2015). AMR has become a critical global health concern. Of particular worry is antibiotic-resistant *S. aureus*, a highly adaptable bacterium causing a range of illnesses, from skin infections to severe systemic disorders like pneumonia, septicemia, bacteremia, and endocarditis (Preeja et al., 2021; Soe et al., 2021). Since the first report of MRSA in the 1960s, MRSA has been acknowledged as a global pathogen of concern (Preeja et al., 2021). Although MRSA infections initially emerged in hospital settings, community outbreaks were documented in Australia and the United States in the 1990s, subsequently spreading worldwide (Chambers, 2001; Mairi et al., 2020). Due to its resistance to various medications, MRSA leads to challenging-to-treat *Staphylococcus* infections. According to the CDC’s 2019 report on antibiotic resistance threats in the United States, MRSA was anticipated to have caused 323,700 cases in hospitalized patients and 10,600 fatalities in 2019, with estimated costs exceeding $1.7 billion in 2017. Adding to the complexity, MRSA can form biofilms, which are crucial for its success in causing infections related to implantable medical devices (Boudet et al., 2021). Due to the emergence of antimicrobial resistance and very slow progress in the discovery of new antibiotics, there is a need for the development of alternative therapies to combat the problem.

Nanotechnology involves harnessing the unique properties of materials that become evident at the nanoscale level. When applied in the realm of medicine and healthcare, it is referred to as nanomedicine, and it has demonstrated its effectiveness in addressing prevalent diseases such as cancer and cardiovascular disorders (Sim and Wong, 2021). The impact of nanotechnology extends across various industries and aspects of society, as it provides products that excel in terms of quality, safety, durability, and intelligence. Even everyday items like sunscreens, cosmetics, sporting goods, tires, and electronics have embraced nanomaterials to enhance their performance (Allhoff et al., 2010). Nanomedicine specifically leverages nanotechnologies within the realm of healthcare (Haleem et al., 2023). In the field of medicine, nanotechnologies hold great promise, spanning diagnostic tools, imaging methods, tissue-engineered constructs, drug delivery systems, implants, and pharmaceutical therapies (Singh and Amiji, 2022). Over the past two decades, there has been a significant shift towards the synthesis of nanoparticles using biological agents, including plants and microbes. These methods are increasingly preferred due to their cost-effectiveness, safety, and environmental friendliness compared to traditional chemical synthesis (Makarov et al., 2014). Using plants for nanoparticle synthesis offers even greater advantages over microbes because cultivating microbes can be relatively costly. Additionally, the use of pathogenic microbes or potentially toxic microbial byproducts can pose additional challenges (Ahmed et al., 2016). On the contrary, plant metabolites, encompassing a range of compounds like amino acids, proteins, enzymes, polysaccharides, tannins, alkaloids, phenolics, terpenoids, saponins, and vitamins, are employed to reduce metal ions and create nanoparticles in an environment-friendly manner (Kulkarni and Muddapur, 2014; Kuppusamy et al., 2016).

Nanotechnology offers a potential solution to the antimicrobial resistance problem. Nanoparticles, particularly silver nanoparticles (AgNPs), show promise in preventing life-threatening infections. They inhibit biofilm formation on medical equipment and have a long history as antimicrobial agents. Despite the toxic effects exhibited by some nanoparticles, nanotechnology, coupled with biosynthesis techniques using plants and microorganisms, can help address toxicity concerns (Ma et al., 2014; Debnath et al., 2019). Silver nanoparticles’ high surface area enhances interaction with microbial cell membranes, effectively targeting bacteria on multiple intracellular fronts and reducing the likelihood of pathogens developing resistance (Chaloupka et al., 2010; Das et al., 2015). The precise mechanism of action of AgNPs remains a subject of ongoing investigation and debate. According to a widely accepted theory, the antimicrobial effect of silver ions can be attributed to their positive charge (Qais et al., 2019b). When silver is in solution or comes into contact with moisture, it releases silver ions from its inert form (Klueh et al., 2000). This release of silver ions leads to an electrostatic attraction between the negatively charged bacterial cells and the positively charged nanoparticles (CaoJin and Mirkin, 2001). This interaction makes AgNPs a promising candidate for bactericidal activity (Eby et al., 2009). Additionally, the production of reactive oxygen species (ROS) is also considered a mechanism underlying the activities of metallic nanoparticles. ROS can cause alterations and fragmentation of cellular proteins, DNA, and lipids, ultimately leading to cell death (Oves et al., 2022).

The *Abroma augusta* Linn is a small evergreen plant native to tropical Asia and a crucial Ayurvedic medicinal plant of the Sterculiaceae family. It has been used to treat various conditions, such as diabetes, joint pain, uterine diseases, and more. This plant is rich in polyphenolic compounds that can reduce, stabilize, and cap metal ions, contributing to the production of stable metal nanomaterials (Mittal et al., 2013). Moreover, the medicinal properties due to the bioactive phytocompounds of *A.* a*ugusta* could impart its effect in the synthesized AgNPs as bioactive compounds are also present on the particle’s surface that act as stabilizing agent. Considering the problem of AMR, it is expected that silver nanoparticles (AA-AgNPs) synthesized using extract of *A*. *augusta* and the bioactive compounds could further enhance the antibiofilm activity of AA-AgNPs. The stabilized AgNPs were characterized using high-resolution transmission electron microscopy (HRTEM), scanning electron microscopy (SEM), and X-ray diffraction studies. The biosynthesized AgNPs demonstrated good antimicrobial and antibiofilm activities against MRSA and VRE.

## 2 Materials and methods

### 2.1 Preparation of leaves extract of *Aborma augusta*

*Abroma augusta* L. leaves were procured from the herbal garden at C.C.S. University in Meerut, Uttar Pradesh, India. The taxonomic identification of the plant was verified by experts from the Department of Botany at Aligarh Muslim University in Aligarh. Leaves of *Abroma augusta* L. were gathered and carefully washed with water. Small leaf sections were then excised and allowed to naturally air dry in a shaded area. Subsequently, the dried leaves were transformed into a powdered form using a grinder. 10 g Abroma augusta L. leaves powder was mixed with 100 mL distilled water. To avoid microbial contamination, the aqueous suspension was prepared in laminar hood under sterile conditions. The mixture was then placed on a magnetic stirrer for a duration of 10 h. Following this, the extract was filtered using Whatman no. 1 paper to remove any particulate matter. To separate denser components, the filtrate was subjected to centrifugation at 8,000 rpm for 7 min. The resulting aqueous extract was then refrigerated at a temperature of 4°C until it was ready for use.

### 2.2 *Aborma augusta* leaf extract-assisted synthesis of AgNPs

Aqueous extract (20 mL) of *Abroma augusta* L. was blended with 1 mM silver nitrate solution (180 mL), resulting in a final volume of 200 mL. The reaction mixture was allowed to incubate at room temperature. As the synthesis of AA-AgNPs progressed, the initial colorless solution of silver nitrate turned to deep brown color that is a distinctive hallmark of AgNPs. However, the reaction mixture was left in a dimly lit environment. To monitor the progress of the reaction, UV-visible spectra were examined across a range of 300–700 nm. To obtain the particles, the suspension was centrifuged at 10,000 rpm for 15 min. Subsequently, the resulting precipitate was re-suspended in water to eliminate any impurities or water-soluble organic residues present at the AgNPs’ periphery. Following this, the biomolecule-coated AgNPs were air-dried at room temperature and subjected to repeated washing with distilled water.

### 2.3 Characterization of green synthesized AgNPs by spectroscopic techniques

#### 2.3.1 UV-visible spectral recording

The optical absorption spectra of AgNPs solution was assessed using an Eppendorf UV-visible spectrophotometer, covering the wavelength range of 300–700 nm. The gathered spectral data was plotted. This technique allows for rapid measurement of the colloidal solution’s particle characteristics, and it doesn’t necessitate any calibration (Qais et al., 2019b).

#### 2.3.2 Fourier-transform infrared spectroscopy

In order to identify potential functional groups of *Aborma augusta* L. extract that might contribute to the capping and effective stabilization of the produced AgNPs, Fourier-transform infrared (FTIR) spectroscopy was employed. For the purpose of creating pellets, AA-AgNPs were mixed with KBr (1:100). Using a Perkin-Elmer FTIR-100 Spectrophotometer, the FTIR spectrum spanning the range of 400–4,000 cm^−1^ was recorded in the diffuse reflectance mode at a resolution of 2 cm^−1^ (Qais et al., 2018). Various peaks obtained in FTIR data were analyzed by comparing them with control.

#### 2.3.3 X-ray diffraction analysis

The X-ray diffraction (XRD) pattern of AA-AgNPs was acquired utilizing the Rigaku Miniflex-II Desktop X-ray Diffractometer with Cu_Kα radiation (*λ* = 1.54060 Å) and a nickel monochromator, operating at a voltage of 30 kV. The pattern was recorded over a 2θ range spanning from 5° to 80°. The average crystal size of the WS-AgNPs was determined through the application of Debye–Scherrer’s equation.

$$D=\frac{k\lambda }{\beta \cos\theta }$$

where *D* is the average crystal size of AA-AgNPs, *K* is the constant of Debye–Scherrer’s equation, *λ* is the wavelength of the X-ray source used, and β is full width at half maximum of the diffraction peak.

#### 2.3.4 Scanning electron microscopy (SEM) and energy dispersive X-ray (EDAX)

In order to discern the surface morphology of the nanoparticles, a thin layer of AA-AgNPs was evenly deposited onto glass coverslips to form films. Subsequently, a gold coating sputter was applied to the samples. For this purpose, electron microscope from JEOL (JSM6390LV, Japan) along with the WINDOW BAS software, was employed. The selected area electron diffraction (SAED) pattern was utilized to elucidate the composition of the biosynthesized AA-AgNPs. Additionally, energy-dispersive X-ray spectroscopy (EDX) was employed to identify the elemental constituents present within the AA-AgNPs.

#### 2.3.5 Transmission electron microscopy (TEM)

The morphological configuration of the synthesized AA-AgNPs was examined through TEM analysis using a JEM-2100, JEOL, Japan. The electron microscope was operated at 200 kV. For this examination, a droplet of the suspension of AA-AgNPs was placed onto a carbon-coated copper grid, serving as a substrate for TEM assessment.

#### 2.3.6 Zeta potential measurement

The assessment of Zeta potential was conducted using a Zeta sizer (Malvern Zeta sizer NanoZS 1070) equipped with zeta cells and polycarbonate cells containing gold-plated electrodes. The zeta potential was evaluated utilizing water as the medium for sample preparation. In order to achieve the desired light scattering behavior from the AgNPs, dried biosynthesized AA-AgNPs powders were combined with water (Singla et al., 2022).

### 2.4 Bacterial isolates

Clinical isolates were sourced from samples including pus, urine, fluids, and blood, which were sent to the Department of Microbiology from the outpatient and in-patient departments of JN Medical College and Hospital in Aligarh. These organisms were cultivated and isolated using a standard medium, and the isolated colonies underwent identification through conventional biochemical techniques and the Vitek-2 automated system (Biomerieux, France) employing VITEK 2 ID cards. Antibiotic susceptibility was conducted by employing the Kirby-Bauer disc diffusion method, which involved the use of commercial antibiotic discs sourced from Hi-media (India). This testing procedure was conducted in strict adherence to the guidelines outlined in the CLSI M100-S30, ensuring standardized and reliable results (CLSI, 2021). A total of 10 isolates each yielding methicillin-resistant *S. aureus* (MRSA) and vancomycin-resistant enterococci (VRE), and 1 isolate each yielding methicillin-sensitive *S. aureus* and vancomycin-sensitive enterococci, were included in the study. As a control, the standard strain of *S. aureus* ATCC 25923 was employed. The confirmation of VRE was additionally conducted through PCR for van A and van B as per standard protocol (Samadi and Asgharzadeh, 2014).

### 2.5 Antibacterial activity of AA-AgNPs

The antimicrobial property of the AA-AgNPs, derived from *Abroma augusta* L. leaf extract, against the pathogenic MRSA and VRE was evaluated through the well diffusion technique (Ibrahim, 2015). Various methodologies were also employed to assess the antibacterial effectiveness. The microorganisms were cultured on nutrient agar plates and incubated at 37°C. Subsequently, a sterile cotton swab was employed to spread each culture onto individual Muller Hinton agar plates. The wells of 6 mm diameter were punched in the agar plates and then 80 µL AA-AgNPs suspension from 100 μg/mL stock solution was placed into the wells. For the bacterial colonies’ growth, the cultures were allowed to develop over a 24 h period. The Kirby-Bauer method was employed to measure the zone of inhibition. Controls were established using distilled water. The antibacterial efficiency was estimated based on the size of the inhibition zone (measured in mm) surrounding the wells. The experimentation process was replicated thrice, and the obtained average outcome was utilized for further analysis.

### 2.6 Determination of minimum inhibitory concentration (MIC)

The broth dilution method was employed to ascertain the MIC, the minimum concentration to AA-AgNPs necessary to halt the growth of a specific strain of bacteria (Polash et al., 2022). Alongside an adjusted bacterial concentration (0.5 McFarland’s standard), the AA-AgNPs concentrations spanned from 2 to 128 μg/mL. Within the study, the positive control involved Mueller Hinton broth medium with the bacteria under examination, while the negative control comprised solely of inoculated broth. The assay was conducted in 96-well polystyrene plates. The temperature was maintained at 37°C, and incubation duration was 24 h. A total of six test sets were conducted to validate the MIC value for the target bacteria. The visual cloudiness of the tubes before and after incubation was used as a determinant of the MIC. After observing the bacterial growth, 100 μL aliquots were extracted from all tubes indicating no visible bacterial growth and plated onto Mueller Hinton agar plates. These plates were devoid of AA-AgNPs and were subsequently kept at 37°C for 24 h. The examination of MBC (minimum bactericidal concentration) was performed before and after incubation to ascertain the presence or absence of bacterial growth on the agar plates. The MBC outcome signifies the lowest concentration of the antimicrobial agent required to eliminate 99.9% of the initial bacterial population.

### 2.7 Interaction of AA-AgNPs with bacterial cells examined by HR-TEM

The interaction between AA-AgNPs and MRSA/VRE was observed utilizing a HR-TEM operating at 200 kV (FEI Tecnai G2). In this study, one representative strain of each group was used, i.e., VRE-01011901 from VRE group and BAC/1232 from MRSA group. For the experiment, MRSA and VRE cells were cultured in nutrient broth for a 24 h period at 37°C. After the incubation, the culture was subjected to centrifugation, and the resulting pellet was reconstituted in 1X PBS (phosphate-buffer saline). Post-dissolution in PBS, the pellet was exposed to 16 μg/mL of AA-AgNPs and subsequently incubated at 37°C for another 24 h. Following this incubation, the culture underwent centrifugation once more, and the supernatant was discarded. The pellet was washed three times with PBS, then fixed in 2.5% glutaraldehyde for a duration of 4 h at 4°C. After this fixation, a series of graded alcohols (30%, 50%, 70%, and 90%) were used for triple rinsing, preparing it for drying. Subsequently, the pellet was immersed in white resin overnight to facilitate bonding (Ali et al., 2018). Following these procedures, drops of the treated bacterial cells with AA-AgNPs and the control counterparts were separately placed on coverslips, setting the stage for the final examination in TEM and SEM.

### 2.8 Biofilm inhibition assays

#### 2.8.1 Quantitative examination of biofilms using microtiter plate method

The capacity of AA-AgNPs to inhibit the biofilm formation at sub-MICs against VRE and MRSA was assessed in polystyrene microtiter plate employing crystal violet as the staining agent (Qais et al., 2021). In brief, 1% of the overnight cultures of the targeted bacteria were introduced into 96-well microtiter plates, each containing 100 μL of fresh LB broth, with or without nanoparticles (at sub-MICs). The microtiter plate was then incubated overnight at 37°C. Subsequent to the incubation period, free-floating cells were eliminated, and the wells underwent a thorough triple wash using sterile PBS. The plate was subsequently left at room temperature for 30 min. The biofilm was subjected to staining using crystal violet (0.1% w/v) for a duration of 10–15 min, followed by rinsing with sterile PBS to eliminate any unbound crystal violet. Finally, the dye affixed to the cells was dissolved in 200 μL of 95% ethanol to each well, and the biofilm was quantified using a microplate reader at 620 nm.

The percentage inhibition of biofilms was calculated utilizing the subsequent formula.

$$Percentage\;of\;inhibition=\frac{\left({Control\;OD}_{620nm}-{Test\;OD}_{620nm}\right)}{{Control\;OD}_{620nm}}\times 100$$

#### 2.8.2 Detection of biofilm inhibition by AA-AgNPs using confocal laser scanning microscopy (CLSM)

Further analysis of biofilm inhibition was carried out utilizing CLSM following the earlier described procedure (Banas et al., 2001). In this study, one representative strain of each group was used, i.e., VRE-01011901 from VRE group and BAC/1232 from MRSA group. To a 12-well microtiter plate fitted with glass coverslips, 100 μL of MRSA and VRE was inoculated. Each well contained NB broth that was challenged with varying concentrations of AA-AgNPs. Subsequent to inoculation, the plate was incubated at 37°C for a duration of 24 h. Upon the completion of the incubation period, the crystal coverslips were rinsed with PBS and then subjected to staining. For this staining process, the coverslips were treated with 15 M propidium iodide (PI) for 15 min at room temperature. This allowed the identification of dead bacterial cells, which were distinguished by their red color. After the 15-minute staining, the cells were once again rinsed with PBS and subsequently treated for another 15 min with 50 g/mL concanavalin-A-conjugated fluorescein isothiocyanate (ConA-FITC). This was employed to stain the glycocalyx matrix green. PI was excited at 520 nm, and its emission was measured at 620 nm. Similarly, ConAFITC was excited at 495 nm, and its emission was measured at 525 nm. The bacteria were visualized using Fluoview FV1000 Espectral Olympus CSLM (Olympus Latin America, Miami, FL, United States) equipped with a UPlanSApo 100/1.40 oil UIS2 Olympus oil immersion lens. The images were captured from randomly selected sites, each image was then carefully scrutinized utilizing an Olympus Fluoview FV 1000.

## 3 Results and discussion

### 3.1 Synthesis and characterization of Aa-AgNPs

**FIGURE 1:**
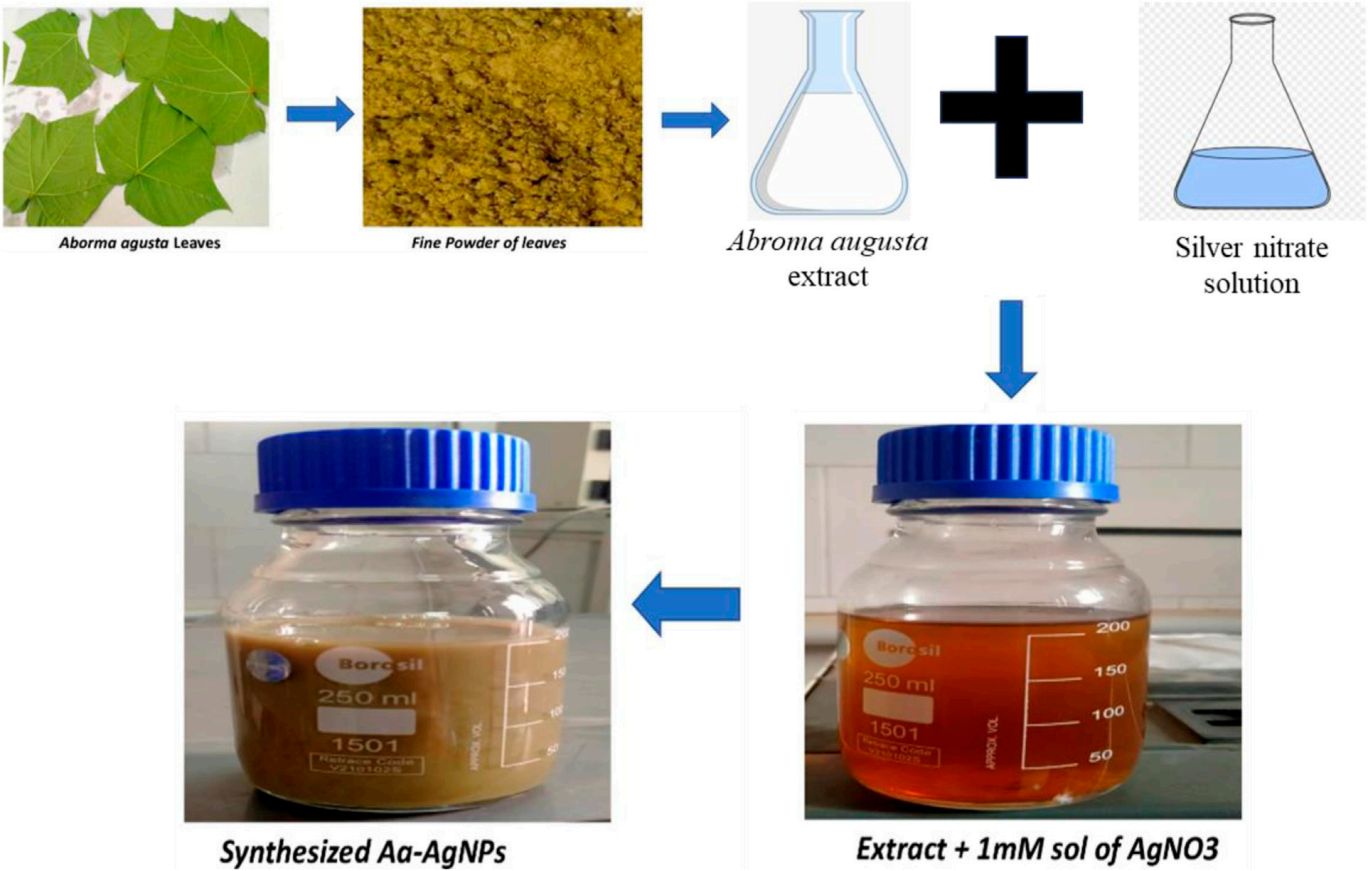
Diagrammatical representation of formation of AA-AgNPs from *Aborma augusta* leaves.

Figure 1 illustrates the schematic depiction of the nanoparticle production process. The addition of A. augusta extract induced a notable change in the color of silver nitrate, aligning with the activation of surface plasmon resonance. The silver nitrate solution was colorless, however the addition of *A. augusta* extract resulted in the change of color from transparent to dark brown. This color shift signified the synthesis of AA-AgNPs. Nanoparticles possess unique optical characteristics that are responsive to factors like size, shape, aggregation, concentration, and refractive index at their surface.

#### 3.1.1 UV-vis spectral analysis

**FIGURE 2:**
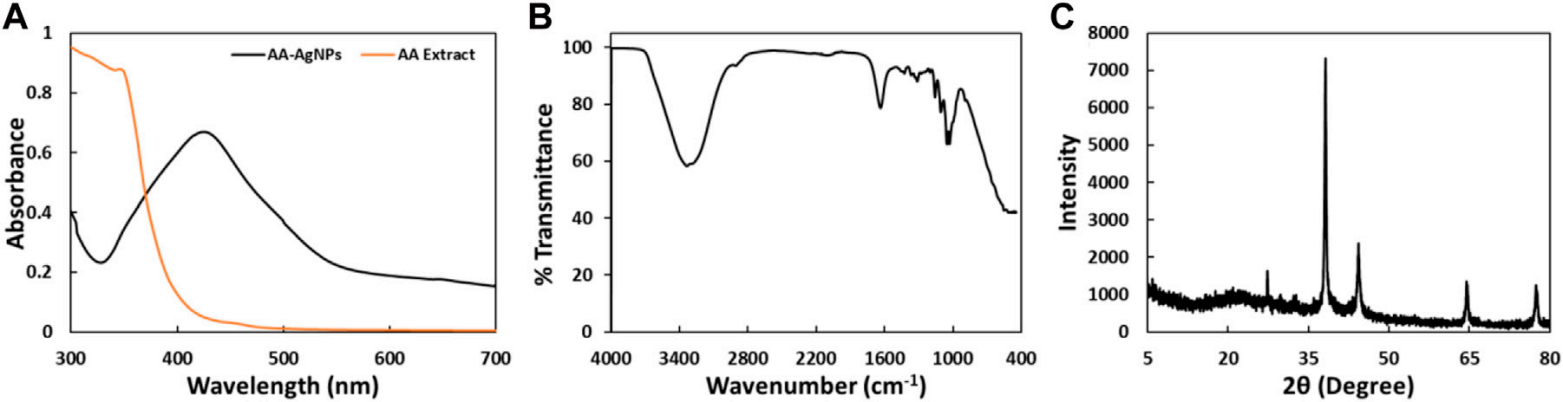
**(A)** UV-Vis spectra of AA-AgNPs synthesized using the leaf extract of *Aborma augusta* and leaf extract of *Aborma augusta* in the range of 300 nm–700 nm. **(B)** FTIR spectrum of AA-AgNPs synthesized using the leaf extract of *Aborma augusta*. **(C)** XRD pattern of AA-AgNPs synthesized using the leaf extract of *Aborma augusta*.

The tool is used for preliminary characterization of nanomaterials. UV-vis spectroscopy stands as a straightforward, rapid, sensitive, and discerning technique used to evaluate fundamental traits across a wide spectrum of synthetic nanoparticles. It is among the methods employed to identify the development of nanoparticles within an aqueous solution. In this investigation, a peak at 424 nm was identified after 24 h of synthesis. The UV-Vis spectra of AgNPs synthesized using leaf extract of *Aborma augusta* (AA extract) is shown in Figure 2A. Similar findings were reported earlier (Qais et al., 2019a) in which synthesized AgNPs from *Carum copticum* extract and noted that the distinct absorption peak of AgNPs in UV-vis spectra emerged roughly at 450 nm.

#### 3.1.2 FTIR spectral analysis

The FTIR spectrum of the biosynthesized AA-AgNPs produced using *Aborma augusta* leaf extract is depicted in In Figure 2B. Various vibration modes were identified that ascertain the presence of functional groups within the AA-AgNPs. Notably, the IR peaks corresponding to 3336, 1638, 1429.3, 1316.2, 1160.8, 1109.8, 1055.2, and 1032.4 cm^−1^ were detected in AA-AgNPs (Table 1). A broad stretching at 3336 cm^−1^ is indicator of presence of hydroxyl residue (Nagaraju et al., 2017). An additional significant stretching vibration appears within the range of 1638 cm^−1^ is attributed to the amide groups in proteins and aromatic compounds containing benzene rings. Moreover, it is noteworthy for its relevance to phenolic and flavonoid compounds, as they also display a pronounced vibration at this specific wavenumber (Prathna et al., 2011). The presence of a peak at 1316 cm^−1^ signifies the stretching vibrations of C-N within the aromatic amines (Mahmood et al., 2021). The asymmetric stretching vibrations occurring at 1160 cm^−1^ are associated with the C–O–C bond (Rabbani et al., 2019). Additionally, the FTIR spectrum of *Aborma augusta* leaf extract was taken (data not shown). It was interesting to note that there were certain peaks that are common in FTIR spectra of nanoparticles and the extract. This indicates the presence of some phytocompounds or functional groups of *Aborma augusta* in AA-AgNPs.

**TABLE 1:** FTIR spectra of AA-AgNPs revealed many peaks and their corresponding functional groups.

S. No.	Absorption peak (cm^−1^)	Functional group
1	3336	N–H aliphatic primary amine
2	1638	C= C alkene
3	1429.3	C=H alkane
4	1316.2	C –N amine
5	1160.8	C–O aliphatic ether
6	1109.8	S – O sulfoxide
7	1055.2	S –O amine
8	1032.4	S=O sulfoxide

#### 3.1.3 XRD pattern analysis

The confirmation of the crystalline structure of the produced AA-AgNPs was accomplished through the identification of peaks at 38, 44, 64, and 78 nm as illustrated in Figure 2C. These peaks corresponded to the (111), (200), (220), and (311) facets of the crystal planes. The experimental XRD data was compared with the standard JCPDS file 04–0783, showcasing angles that exhibited Bragg’s reflections. These reflections were indexed by employing data obtained from the face-centered cubic structure of silver (Rajawat and Qureshi, 2014). The average particle size calculated using Debye–Scherrer’s equation was found to be 25.36 nm.

#### 3.1.4 Scanning electron microscopy (SEM) and energy dispersive X-ray (EDAX)

**FIGURE 3:**
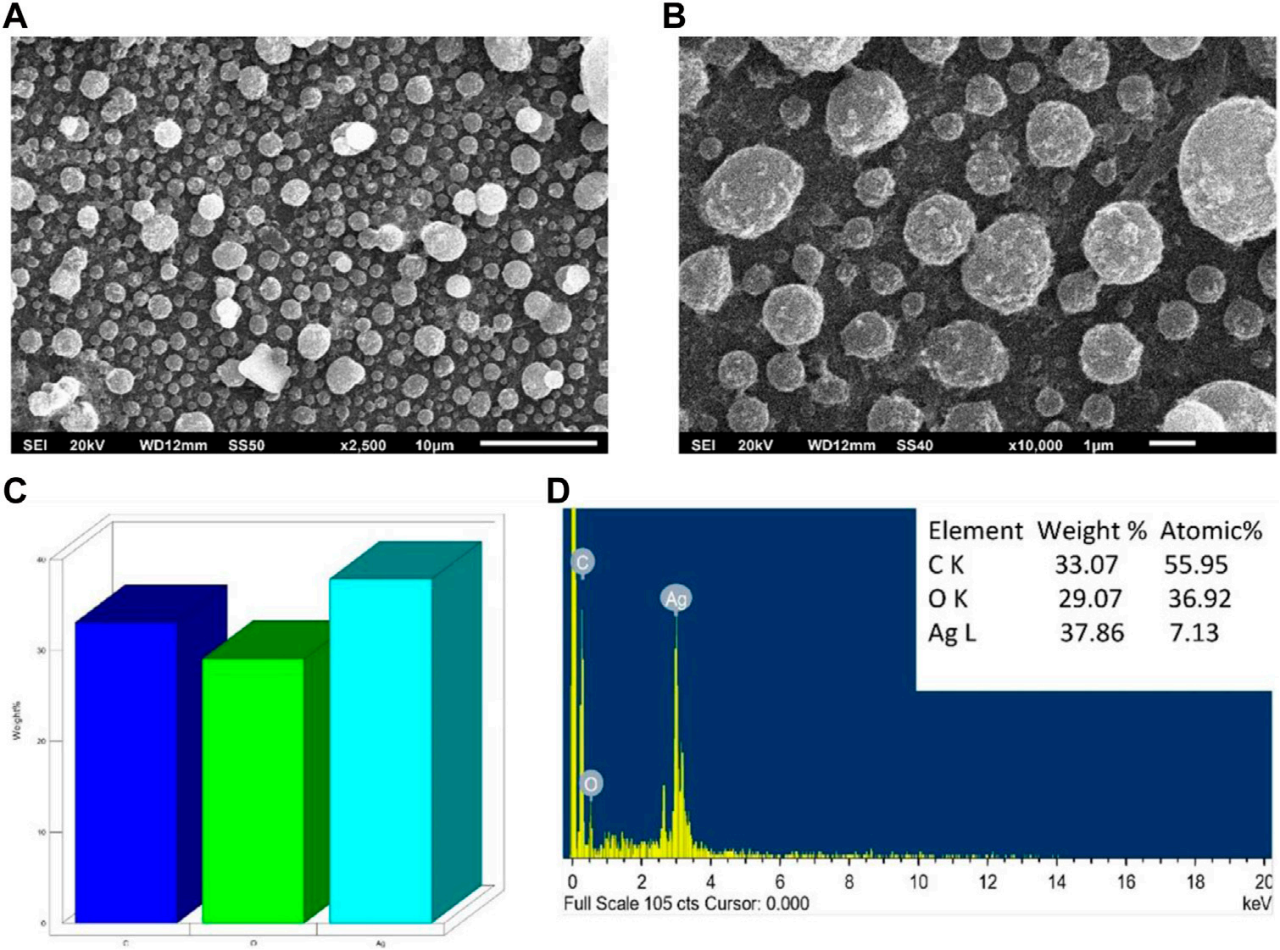
SEM analysis of AA-AgNPs made from an aqueous extract of *Aborma augusta* using green synthesis. **(A)** SEM micrograph at 2,500 magnification. **(B)** SEM micrograph at ×10000 magnification. **(C)** Weight percentage of different elements in AA-AgNPs. **(D)** EDX spectrum of AA-AgNPs.

The morphology, size, and elemental composition of the powdered AA-AgNPs were examined using SEM coupled with EDAX. Images were captured at magnifications of 2,500 and X100,00 to provide a more distinct depiction of the nanoparticles (Figures 3A, B). The examination revealed that the nanoparticles exhibited dimensions ranging between 13 and 23 nm (Chauhan et al., 2016). The presence of silver signals was confirmed through the analysis by an EDS spectrometer (Figures 3C, D). The silver signal, with a peak at three keV, substantiated the presence of elemental silver. Additionally, weaker signals were detected for other elements like oxygen and carbon, attributed to the presence of confined surface plasmons. The composition of carbon, oxygen, and silver by weight percent were 33.07, 29.07, and 37.86%, respectively.

#### 3.1.5 Transmission electron microscopy (TEM)

**FIGURE 4:**
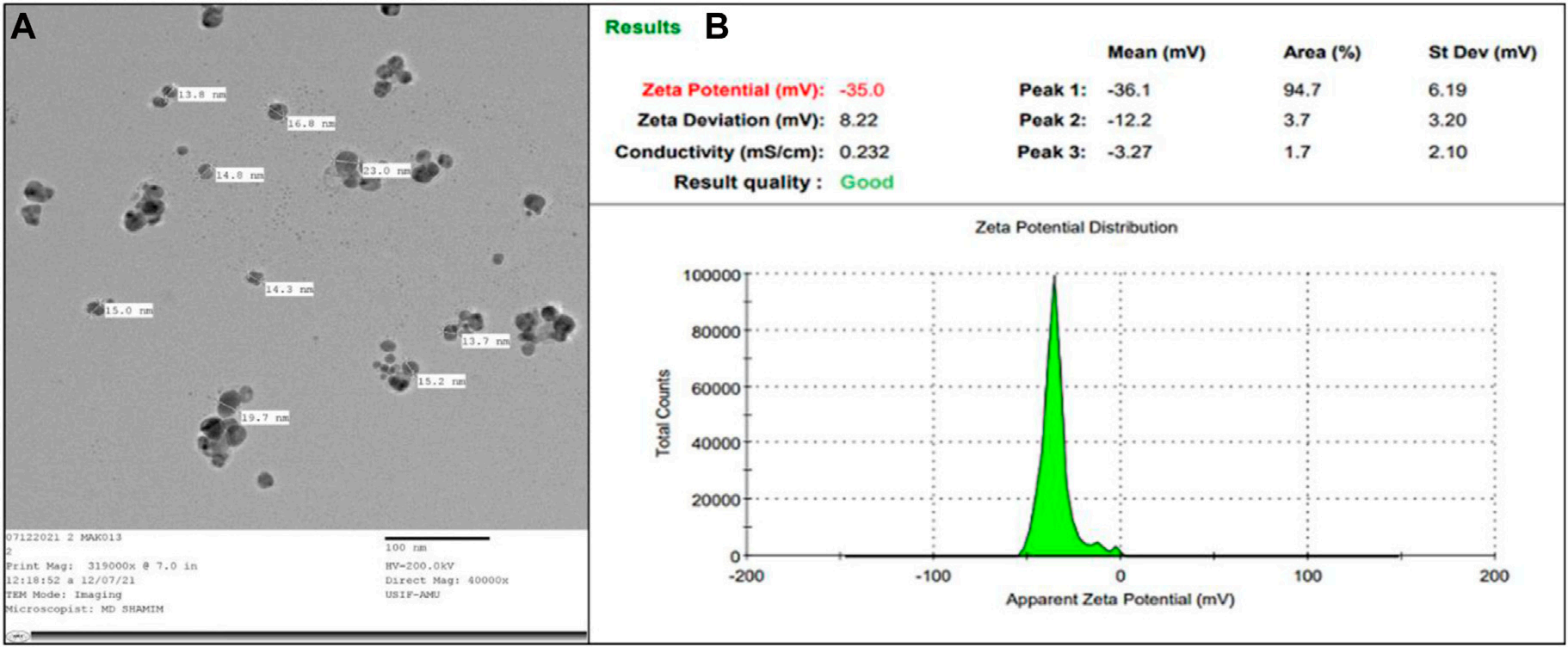
**(A)** TEM of AA-AgNPs made from an aqueous extract of *Aborma augusta* at ×40000 magnification. **(B)** The zeta potential of synthesized AA-AgNPs.

TEM images were employed to ascertain the shape and dimensions of AA-AgNPs, as illustrated in Figure 4A. The dispersed particles exhibited a spherical configuration, revealing a substantial surface area of the created nanostructures. The synthesized nanoparticles exhibited a size range spanning from 13 to 23 nm, with an average diameter of 18 nm (Sadeghi et al., 2015). The size of AA-AgNPs obtained from TEM is also in agreement of XRD data. Upon examination, the TEM images displayed a consistent homogeneity in diameter and a spherical shape.

#### 3.1.6 Zeta potential

The robust zeta potential of the nanoparticles reached its maximum at −35 mV, as depicted in Figure 4B. These findings indicated that the nanoparticle’s surface exhibited a negative charge, extending throughout the surrounding medium (Mukherjee et al., 2014). The observed negative charge on the AA-AgNPs confirmed their mutual repulsion, underscoring their lack of attraction to each other. The negative charge of the nanoparticles contributes to their heightened stability (Krishnakumar et al., 2018).

### 3.2 Antibacterial activity of AgNPs

Table 2 demonstrates the antibacterial effects of AA-AgNPs against both susceptible strains and drug-resistant human bacterial isolates such as MRSA and VRE. Across all tested microorganisms, the range of inhibition zones ranged from 12 to 26 mm. Notably, the zones were wider in susceptible and standard strains, observed in both *S. aureus* and *E. faecalis*, as compared to drug-resistant strains. The heightened antimicrobial potency of AA-AgNPs could be attributed to their expansive surface area, enabling improved interaction with microorganisms. Additionally, a synergistic effect arises when these particles amalgamate with other natural compounds, as discussed in references (Logeswari et al., 2015; Durán et al., 2016). In another, it was demonstrated that the combination of AgNPs and phenazine-1-carboxamide amplified the antibacterial impact against methicillin-resistant *S. aureus* strains by a factor of 32 (Cardozo et al., 2013). This augmentation resulted in notable morphological changes to the bacterial cell wall. The mechanism of action involves targeting the respiratory chain, impeding cell division, and compromising the bacterial membrane, ultimately causing cell demise. Furthermore, the nanoparticles facilitate the release of Ag^+^ within bacterial cells, thereby intensifying the bactericidal efficacy (Morones et al., 2005).

**TABLE 2:** Zone of growth inhibition of test bacteria by AA-AgNPs.

Lab reference no.	Bacterial isolates	Zone of inhibition in (mm)
*ATCC*	*Enterococcus faecalis ATCC*	24
02,081,907	*Enterococcus faecalis*	26
01,011,901	*VRE*	16
26,101,801	*VRE*	18
31,011,910	*VRE*	16
18,021,904	*VRE*	20
18,021,905	*VRE*	14
18,021,906	*VRE*	16
18,021,907	*VRE*	16
18,021,908	*VRE*	12
18,021,912	*VRE*	16
18,021,909	*VRE*	18
*ATCC*	*S. aureus ATCC*	26
PUS/4829	*Staphylococcus aureus*	18
BAC/1232	*MRSA*	20
PUS/4230	*MRSA*	18
PUS/4331	*MRSA*	14
BAL/4367	*MRSA*	16
PUS/4364	*MRSA*	16
BAC/920	*MRSA*	14
BAC/921	*MRSA*	18
PUS/3347	*MRSA*	14
PUS/2981	*MRSA*	16
PUS/1220	*MRSA*	12

### 3.3 HR-TEM shows the impact of AA-AgNPs on bacterial cells

**FIGURE 5:**
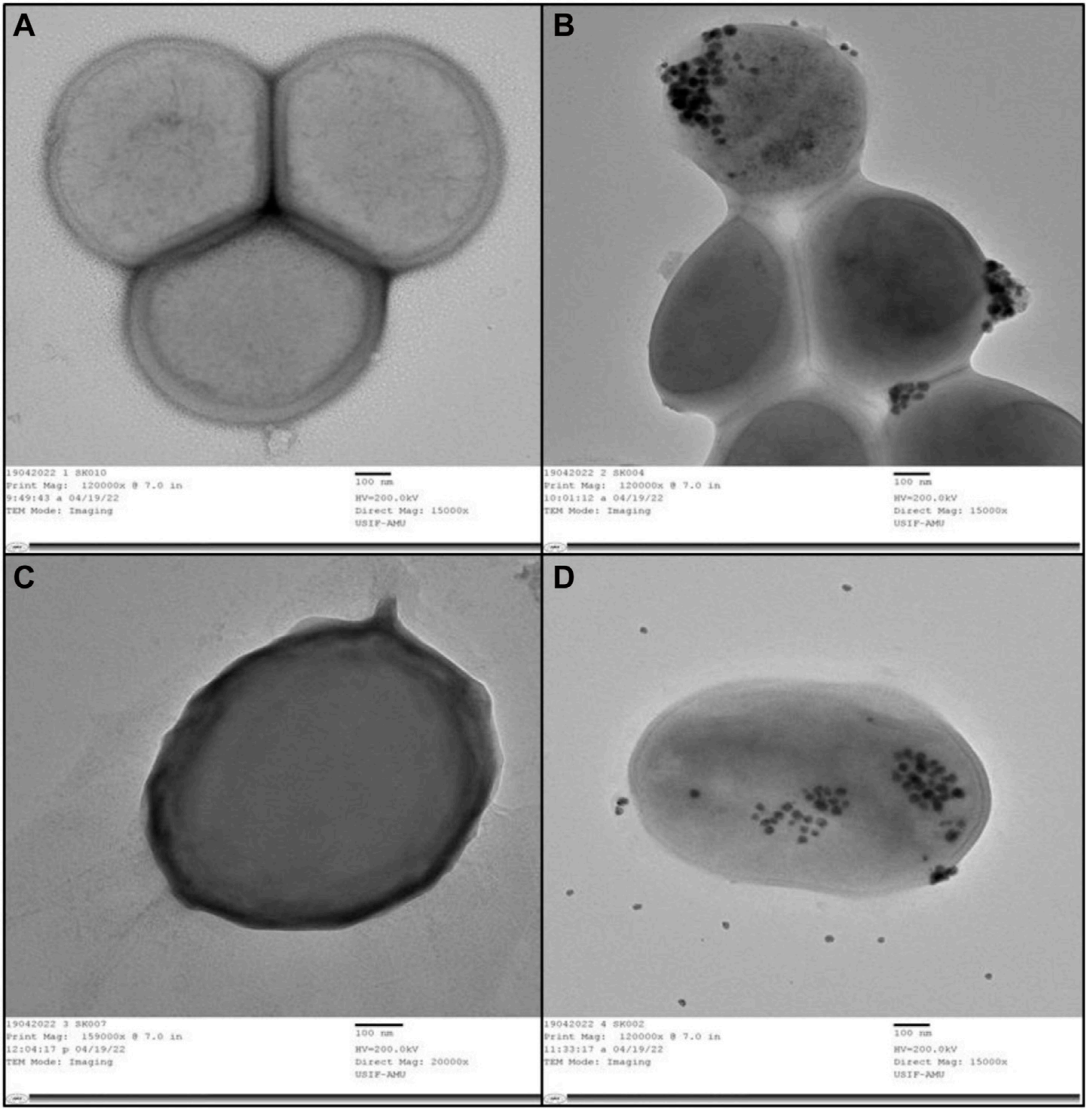
**(A)** TEM of internal and outer structures of untreated MRSA that appeared to be normal. **(B)** TEM of MRSA treated with AA-AgNPs at 16 μg/mL in which bacterial cells was observed to be damaged. **(C)** TEM of internal and outer structures of untreated VRE appeared that appeared to be normal. **(D)** TEM of VRE treated with AA-AgNPs at 16 μg/mL in which bacterial cells was observed to be damaged.

The internal structures of untreated MRSA and VRE displayed their normal composition, featuring an intact outer membrane and cytoplasmic membrane (Figures 5A, C). Following exposure to AA-AgNPs at 16 μg/mL, a notable disruption occurred in the bacterial cells, portraying substantial damage (Figures 5B, D). In this altered state, there was visible leakage of the cell’s internal components. Alternatively, under the influence of the same concentration treatment, the cell wall ruptured, permitting the entry of nanoparticles from various points. This incursion led to significant cellular damage and ultimately culminated in cell death. A study examined the effect of AgNPs on the bacterial morphology of *E. coli*, *S. typhimurium*, and *S. aureus*. They found that AgNPs disrupt the cell membrane of *E. coli* and *S. typhimurium*. AgNPs did not caused any apparent damage to *S. aureus*. However, sublethal concentrations in presence of chloramphenicol and kanamycin showed a synergistic and an additive effect in which dramatic cellular damage was recorded (Vazquez-Muñoz et al., 2019).

### 3.4 SEM examination of morphological changes after AA-AgNPs treatment

**FIGURE 6:**
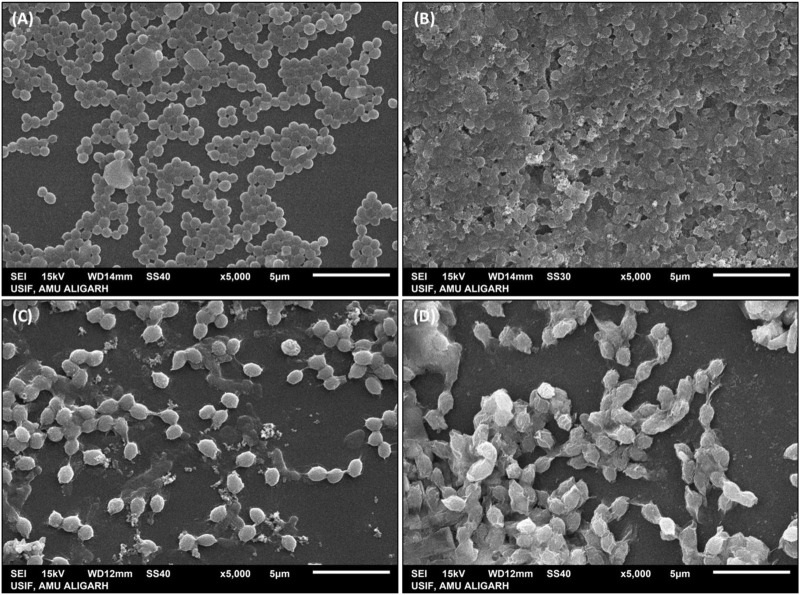
SEM images of **(A)** untreated MRSA, **(B)** MRSA treated with 16 μg/mL AA-AgNPs, **(C)** untreated VRE, and **(D)** VRE treated with 16 μg/mL AA-AgNPs.

The interaction between Aa-AgNPs and MRSA/VRE cells was examined through SEM. With time, the cells subjected to AA-AgNPs treatment, both MRSA and VRE, displayed an escalating extent of damage to their cell walls (Figures 6B, D). In contrast, untreated cells exhibited no indications of damage, thus providing compelling evidence of AA-AgNPs’ bactericidal effect (Figures 6A, C) (Marslin et al., 2015). The primary contributor to these morphological abnormalities lies in the degradation of the bacterial cell membrane following exposure to AA-AgNPs. AA-AgNPs have the potential to induce harm through various mechanisms, including the dismantling of bacterial cell components, rupture of the cell membrane, disruption of the microbial respiration control system, or penetration into the cytoplasm (Dror-Ehre et al., 2009). Earlier, SEM images of *S. aureus* and *E. coli* showed that AgNPs interact with bacterial cells at a sublethal dose and such interaction led to the binding of AgNPs to the cell surfaces and caused cellular damage (Ali et al., 2015).

### 3.5 Determination of minimum inhibitory concentration (MIC)

The MIC of AA-AgNPs against MRSA and VRE isolates ranged from 8 to 32 μg/mL. The MIC was determined to be 16 μg/mL for *Enterococcus faecalis* ATCC and 8 μg/mL for sensitive *Enterococcus faecalis*. However, the MIC of AA-AgNPs against MRSA-sensitive and standard strains of *S. aureus*, the MIC ranged between 8 and 32 μg/mL (Table 3). Our discovery aligns with a previous study in which the MIC and MBC values of AgNPs produced from *Eucalyptus globulus* extract against MRSA and MSSA were calculated as 27 and 30 μg/mL, and 30 and 33 μg/mL, respectively (Ali et al., 2015). Similarly, another study reported the MIC of AgNPs prepared from *Withania somnifera* extract against *S. aureus* to be 32 μg/mL (Qais et al., 2018).

**TABLE 3:** Minimum inhibitory concentration (MIC) of test bacteria by AA-AgNPs.

Lab reference number	Bacterial strains	MIC value (µg/mL)
*ATCC*	*Enterococcus faecalis ATCC*	16
02,081,907	*Enterococcus faecalis*	8
01,011,901	*VRE*	32
26,101,801	*VRE*	16
31,011,910	*VRE*	16
18,021,904	*VRE*	8
18,021,905	*VRE*	8
18,021,906	*VRE*	32
18,021,907	*VRE*	16
18,021,908	*VRE*	8
18,021,912	*VRE*	8
18,021,909	*VRE*	8
*ATCC*	*S. aureus ATCC*	16
PUS/4829	*Staphylococcus aureus*	16
BAC/1232	*MRSA*	32
PUS/4230	*MRSA*	8
PUS/4331	*MRSA*	8
BAL/4367	*MRSA*	16
PUS/4364	*MRSA*	32
BAC/920	*MRSA*	32
BAC/921	*MRSA*	8
PUS/3347	*MRSA*	32
PUS/2981	*MRSA*	16
PUS/1220	*MRSA*	8

### 3.6 AA-AgNPs inhibits the biofilm of test bacteria

#### 3.6.1 Quantitative analysis of biofilm inhibition by microtiter plate method

**FIGURE 7:**
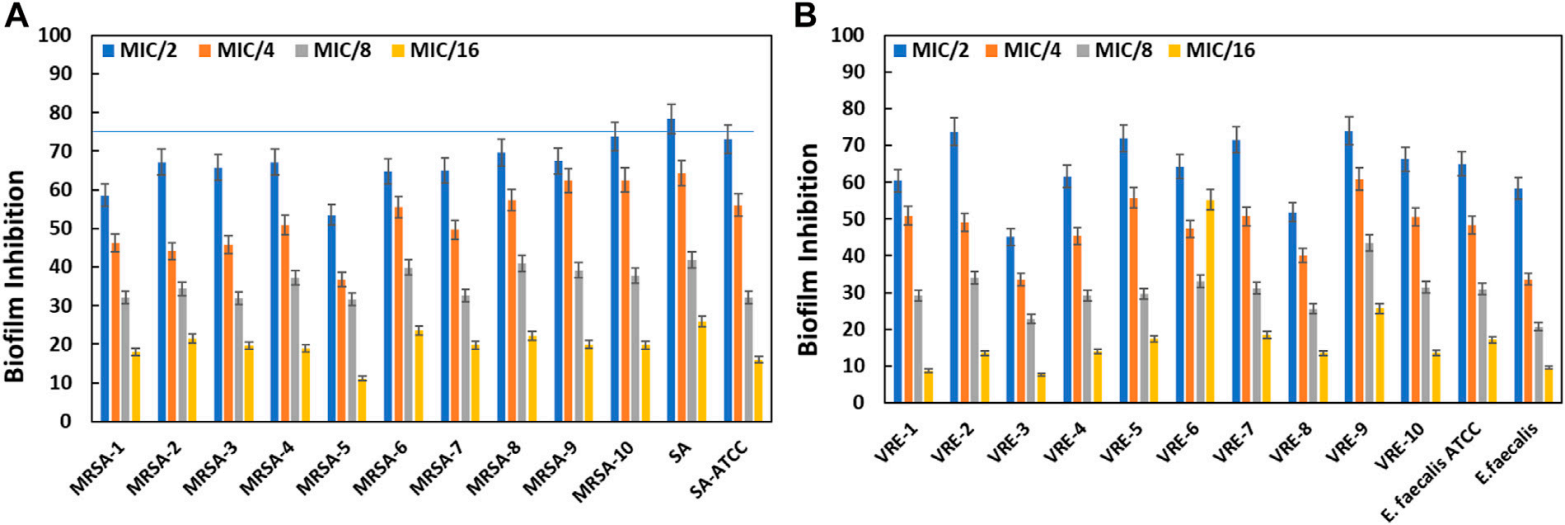
Inhibition of biofilms of MRSA by AA-AgNPs **(A)** and VRE by AA-AgNPs **(B)**. Results are shown in triplicate with standard deviation (SD).

The objective of this assay was to explore the efficacy of AA-AgNPs in curtailing biofilm formation of various bacterial strains, at sub-inhibitory concentration (sub-MIC). Biofilm formation serves as a common defensive mechanism for bacteria against antimicrobial agents, rendering infections arising from biofilm-forming bacteria challenging to treat. Thus, the pursuit of innovative strategies for inhibiting biofilm formation holds significant importance. The findings unveiled that AA-AgNPs effectively hindered biofilm formation at sub-MICs in all MRSA and VRE bacterial strains (Figure 7). At varying sub-MICs, the particles were capable of obstructing biofilm development by more than 75% in specific bacterial strains. The effect of AA-AgNPs on the biofilms of test bacterial pathogens was found to be dose dependent. Notably, the particles exhibited distinct effects on different bacterial types, with varying degrees of sensitivity. Overall, the outcomes indicate that nanoparticles offer a promising avenue to thwart biofilm formation across multiple bacterial strains, even below the MIC threshold. Such findings could hold significant implications for the development of novel antimicrobial agents aimed at combating infections linked to biofilms.

The significance of biofilms in medical contexts stems from their contribution to bacterial pathogenesis and the challenge they pose for complete eradication using antibiotics (Hall and Mah, 2017). The process of biofilm formation is a highly organized sequence of events, closely regulated by bacterial cellular communication (Qin et al., 2015). Roughly, around 80% of infections are connected to biofilm development, and the persistence of these biofilms is closely tied to the composition of the matrix, primarily composed of EPS, proteins, lipids, and eDNA (Flemming and Wingender, 2010). The outcomes of this study align with prior findings, where silver nanoparticles synthesized using green methods reduced *S. aureus* biofilms by more than 40% at half sub-inhibitory dose (Qais et al., 2018). Within the biofilm matrix, there are water channels that facilitate nutrient transportation, which also allows particles to diffuse and exhibit anti-biofilm activity. Furthermore, nanoparticles have been documented to attach to and penetrate bacterial membranes, accumulate within bacterial cells, and ultimately induce bacterial death (Ansari et al., 2014).

#### 3.6.2 Confocal laser scanning microscopy

**FIGURE 8:**
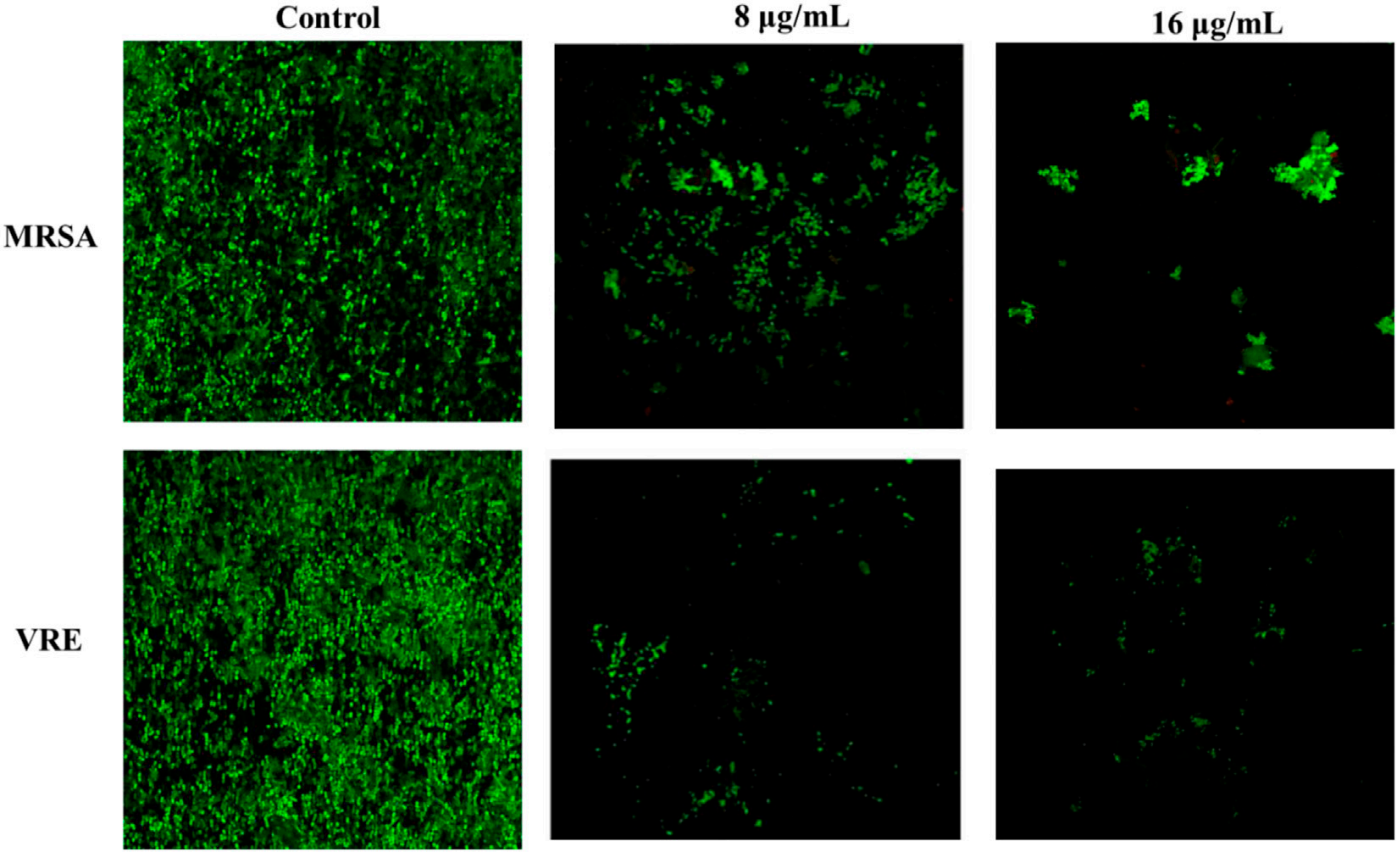
Confocal laser scanning microscopic images of MRSA and VRE in the absence and presence different concentrations of AA-AgNPs.

Propidium iodide was employed to identify bacterial cells, which became easily distinguishable due to their size and shape. In various applications, time-lapse microscopy conducted with CLSM stands out as an optimal tool for spatial monitoring at a micron-level resolution. This technique enables the non-invasive investigation of biofilms by examining different layers at various depths, thereby facilitating the reconstruction of a three-dimensional structure (Lawrence and Neu, 1999; Psaltis et al., 2007). This method allowed us to assess the impact of AA-AgNPs on the synthesis of the glycocalyx matrix and exopolysaccharides. To achieve this, a dual staining technique involving propidium iodide and ConA-FITC was employed. ConA-FITC, known for its binding to mannose residues, introduced a green staining effect that indicated the presence of a bacterial glycocalyx (Bahey et al., 2019). The presence of dark spots within the biofilm could be attributed to factors such as water movement or variations in the construction of the matrix and exopolysaccharides (Khatoon et al., 2018). By overlaying red and green fluorescence intensities in CLSM images, the formation of the biofilm’s capsular component, extracellular polysaccharides, was affirmed. In the case of untreated MRSA and VRE, the PI stain illuminated bacterial nucleic acids in a fluorescent red color, while the green fluorescence (ConA-FITC) indicated the presence of exopolysaccharides (Figure 8). In control, most of the cells were seen in green color is indicator of live bacterial cells. The number of live cells visible under confocal microscope decreased with the increasing concentration of AA-AgNPs. In the samples treated with AA-AgNPs, the majority of cells were observed to be non-viable as very less exopolysaccharides (green fluorescence) were detected. Biofilms cultivated on coverslips featuring varying concentrations of AA-AgNPs displayed inhibited growth and a limited number of cells exhibiting assorted colony patterns (Figure 8). When AA-AgNPs were introduced, minimal growth accompanied by a sparse cell population was noted, lacking any discernible arrangement pattern. Additionally, the cells displayed morphological distortions as the concentrations of AA-AgNPs increased (Ansari et al., 2014).

## 4 Conclusion

Since the onset of multidrug-resistant bacteria, mortality rate observed over the past two decades due to vancomycin-resistant enterococci is worrisome that poses a notable threat to current medical procedures. Therefore, there is a need for development of alternative strategies to combat this problem. The leaf extracts of *A. augusta* present proved excellent in eco-friendly synthesis of AgNPs and showed potent antibacterial and antibiofilm effects. Apart from preliminary characterization, FTIR results pointed towards the involvement of biological functional groups involved in reduction of silver and their capping and stabilization to form nanoparticles. The particles were crystalline in nature with 25.36 nm in size and negative zeta potential deciphered the stability. AA-AgNPs exhibited robust antibacterial and antibiofilm activities against MRSA and VRE. HR-TEM and SEM analysis provided confirmation that the impact of NP treatment on bacterial morphology was due to membrane rupturing and leakage of intracellular components. AA-AgNPs also demonstrated efficacy in curtailing biofilm formation at the sub-MIC levels. Hence, this study highlights the potential of AA-AgNPs in effectively inhibiting the biofilm formation of MRSA and VRE bacteria. Based on the findings of this research, AgNPs synthesized from *A. augusta* extract could potentially be used in the management of drug resistant microbes. Future studies are warranted to concentrate on ascertaining the optimal dosage and application methods of nanoparticles in clinical scenarios.

## Data Availability

The original contributions presented in the study are included in the article/Supplementary Material, further inquiries can be directed to the corresponding authors.
